# Small Pox

**Published:** 1854-05

**Authors:** 


### SMALL-POX.

There have been two or three fatal cases of small-pox, in this city, within the last few weeks, and the disease is still prevailing to some extent in one or two neighborhoods. Small-pox was prevalent during the last year in several of the eastern cities, and from New York we have a pamphlet by Dr. Joseph C. Hutchinson on the causes of its prevalence there, in which we find the remark, that an inspection of statistical tables "reveals the significant fact that in those countries where vaccination is made compulsory, the proportionate mortality from small-pox is considerably less than half what it is where it is entirely voluntary."

This is a significant fact. If vaccination were universal, we have no doubt that small-pox would be extirpated. In Denmark it had at one time disappeared before the defensive influence of compelled vaccination; but chance and a careless security, says Dr. Watson, have led to its re-introduction there.

There is a disposition in the public mind to question the efficacy of vaccination, which the recurrence of the pest, and especially in vaccinated persons, is calculated to increase. But it should be borne in mind that the operation is often very carelessly and imperfectly performed, from which it must result that the system is not brought under the vaccine influence; and it is further to be remembered that individuals are liable to second attacks of small-pox, and may therefore occasionally suffer with varioloid after having been well vaccinated. But there is no ground for skepticism as to the prophylactic powers of the vaccine virus; on the contrary we are sure that every one who patiently investigates the subject will be ready to adopt the language of Dr. Hutchinson, who declares in closing his laborious inquiry, that his "estimate of the immense value of vaccination is not only undiminished, but is positively increased, and that he feels more inclined to defend from the assaults of ignorance and prejudice the most brilliant triumph of modern medicine."

